# Comparison of the chloroplast genomes and phylogenomic analysis of Elaeocarpaceae

**DOI:** 10.7717/peerj.15322

**Published:** 2023-05-09

**Authors:** Yihui Wang, Yifei Xie, Jiayi Jin, Jinyue Li, Xiangdong Qiu, Yang Tong, Zhongyang Li, Zhixiang Zhang, Wenling Lai

**Affiliations:** 1 School of Life Sciences, Gannan Normal University, Ganzhou, P.R. China; 2 School of Landscape Architecture, Beijing Forestry University, Beijing, P.R. China; 3 Key Laboratory of Nanling Plant Resources Conservation and Utilization, Ganzhou, P.R. China; 4 Center for Integrative Conservation, Xishuangbanna Tropical Botanical Garden, Chinese Academy of Sciences, Xishuangbanna, P.R. China; 5 School of Ecology and Nature conservation, Beijing Forestry University, Beijing, P.R. China

**Keywords:** Elaeocarpus, Chloroplast genome, Divergence time, Phylogenomic

## Abstract

**Background:**

Elaeocarpaceae is a vital family in tropical and subtropical forests. Compared with the important position of Elaeocarpaceae species in forest ecosystem and the concern of medicinal value, the most research on Elaeocarpaceae are classification and taxonomy. Molecular systematics has corrected the morphological misjudgment, and it belongs to Oxalidales. Phylogenetic and divergence time estimates of Elaeocarpaceae is mostly constructed by using chloroplast gene fragments. At present, although there are reports on the chloroplast structure of Elaeocarpaceae, a comprehensive analysis of the chloroplast structure of Elaeocarpaceae is lacking.

**Methods:**

To understand the variation in chloroplast sequence size and structure in Elaeocarpaceae, the chloroplast genomes of nine species were sequenced using the Illumina HiSeq 2500 platform and further assembled and annotated with *Elaeocarpus japonicus* and *Sloanea sinensis* (family Elaeocarpaceae) as references. A phylogenomic tree was constructed based on the complete chloroplast genomes of the 11 species representing five genera of Elaeocarpaceae. Chloroplast genome characteristics were examined by using Circoletto and IRscope software.

**Results:**

The results revealed the following: (a) The 11 sequenced chloroplast genomes ranged in size from 157,546 to 159,400 bp. (b) The chloroplast genomes of *Elaeocarpus*, *Sloanea*, *Crinodendron* and *Vallea* lacked the *rpl*32 gene in the small single-copy (SSC) region. The large single-copy (LSC) region of the chloroplast genomes lacked the *ndh*K gene in *Elaeocarpus*, *Vallea stipularis*, and *Aristotelia fruticosa*. The LSC region of the chloroplast genomes lacked the *inf*A gene in genus *Elaeocarpus* and *Crinodendron patagua*. (c) Through inverted repeat (IR) expansion and contraction analysis, a significant difference was found between the LSC/IRB and IRA/LSC boundaries among these species. *Rps*3 was detected in the neighboring regions of the LSC and IRb regions in *Elaeocarpus*. (d) Phylogenomic analysis revealed that the genus *Elaeocarpus* is closely related to *Crinodendron patagua* on an independent branch and *Aristotelia fruticosa* is closely related to *Vallea stipularis*, forming a clade with the genus *Sloanea*. Structural comparisons showed that Elaeocarpaceae diverged at 60 Mya, the genus *Elaeocarpus* diverged 53 Mya and that the genus *Sloanea* diverged 0.44 Mya. These results provide new insight into the evolution of the Elaeocarpaceae.

## Introduction

In land plants, most chloroplast genomes’ genome is approximately 100–220 kbp in size and has a quadripartite structure, including one large single-copy (LSC) region, one small single-copy (SSC) region, and a pair of inverted repeat (IR) regions (Bock, 2007; Song et al., 2017). These regions are involved in photosynthesis, transcription, and translation, among other functions (Gao, Su & Wang, 2010). With the increase in complete chloroplast genome data, comparative analysis of chloroplast genomes has been widely applied (Wu, 2016). Some lineages, such as ferns (Roper et al., 2007; Karol et al., 2010), gnetophytes (McCoy et al., 2008; Wu et al., 2009), multiple angiosperm families (Goremykin et al., 2003; Cai et al., 2006), and nonphotosynthetic plants (Wicke et al., 2016), have lost some genes. For example, *ycf*1, *ycf*2 and *acc*D have been lost in the family Poaceae (Guisinger et al., 2010), and *rpl*22, *inf*A and *acc*D have been lost in legumes, Lemnoideae, and Acoraceae, respectively (Doyle, Doyle & Palmer, 1995; Goremykin et al., 2005; Wang & Messing, 2011). The *ndh* genes loss events were also detected in heterotrophic plants, utotrophic orchids, gnetophytes and Pinaceae, and pseudogenization occurred in heterotrophic plants (Wakasugi et al., 1994; Wickett et al., 2008; Braukmann, Kuzmina & Stefanovic, 2009; Barrett et al., 2014; Kim et al., 2015; Wicke et al., 2016). In recent years, phylogenomics has shown great advantages in plant phylogenetic research based on chloroplast genomes, providing resolutions for the phylogenies of some taxonomically difficult groups of plants.

Elaeocarpaceae Juss. is a medium-sized family of angiosperms comprising 12 genera and 615 species of trees that grow in tropical and subtropical forests (Coode, 2004; Christenhusz & Byng, 2016). Most studies suggest that Elaeocarpaceae is a sister group to Cephalotaceae and *Brunellia* based on *trn*L-*trn*F and nuclear *ITS* regions (Crayn, Rossetto & Maynard, 2006; Magallon et al., 2015; Harris & Davies, 2016). Heibl & Renner (2012) phylogenetic analysis indicated that Elaeocarpaceae is sister to Cunoniaceae relied on *rbc*L region, *trn*L-*trn*F region and *ITS* region. Molecular phylogenies provide a robust evidence that Elaeocarpaceae has a closest relationship with the clade comprising Cunoniaceae and Cephalotaceae (Phoon, 2015). Besides, the differentiation within Elaeocarpaceae is ambiguous. According to the original research, Elaeocarpaceae diverged in the early Cretaceous (120 Mya) based on *trn*L-*trn*F and nuclear *ITS* regions (Crayn, Rossetto & Maynard, 2006). Combined nuclear and chloroplast DNA sequences, Heibl & Renner (2012) study indicated that Elaeocarpaceae diverged in the late Cretaceous (67 Mya) based on three markers (*rbc*L region, *trn*L-*trn*F region and *ITS* region). Phoon (2015) study also showed that Elaeocarpaceae and its sister lineage (Cunoniaceae + Cephalotaceae) diverged in the late Cretaceous (85 Mya) based on four regions (*psb*A-*trn*H intergenic spcer, *trn*L-*trn*F region, *trn*V-*ndh*C intergenic spcer and nuclear *Xdh*). With the increasing number of genomic data about Elaeocarpaceae on NCBI, it is necessary to conduct comparison of the chloroplast genomes research and divergence time within Elaeocarpaceae (Wang, Zhang & Xie, 2021; Weng et al., 2021). In addition, genetic differentiation within Elaeocarpaceae associated with microsatellite loci is common in many species such as *E. photiniifolia* and *Tetratheca ericifolia* (McPherson et al., 2008; Sugai et al., 2012, 2013; Anthony, Allcock & Krauss, 2016).

Although the taxonomy of Elaeocarpaceae belonged to Oxalidales is widely acknowledged, the relationships within Oxalidales need further study (Savolainen et al., 2000; Soltis et al., 2000; Byng et al., 2016). Furthermore, the age of genera and relationships within Elaeocarpaceae are incongruent in previous studies based on multigene phylogenies (Crayn, Rossetto & Maynard, 2006; Heibl & Renner, 2012; Phoon, 2015), hence the age of the genera within Elaeocarpaceae have not been adequately examined.

Here we used the whole chloroplast genome sequences to further explore phylogenetic relationships within Elaeocarpaceae and other relative families in detail. This study aims to (a) test genetic category between different genus within Elaeocarpaceae, (b) determine the relationships of Elaeocarpaceae within Oxalidales, (c) use the molecular data together with the data from the palaeobotanical literature to infer divergence dates and the biogeographic history of the major clades within Oxalidales and Elaeocarpaceae.

## Materials and Methods

### Plant material and chloroplast genome sequencing

Leaf materials were sampled from nine species representing five genera of Elaeocarpaceae and collected from field in China and the Royal Botanic Gardens (Table 1). Voucher specimens of the collection were deposited at the Museum of Gannan Normal University, Nanling Herbarium (GNNU; Director: Yifei Xie, xie.yifei2018@gmail.com), Museum of Beijing Forestry University (BJFC) and The Royal Botanic Gardens (K). Total genomic DNA was extracted using the magnetic bead method and then sent to Sino Geno Max Company for next-generation sequencing using the Illumina HiSeq (TM) 2500 platform in Beijing, China and the read length generated from the Illumina platform was 150 bp. The raw data were filtered by cutadapt version 1.9.1 and trimmed by Trimmomatic version 0.39 to remove low-quality bases with the parameters in S1 (Martin, 2011; Bolger, Lohse & Usadel, 2014). Then we obtained clean data and uploaded them to the NCBI SRA database in fastq format (Table 2).

**Table 1:** Sampled species of Elaeocarpaceae and their voucher specimens.

No.	Species	Herbarium	Voucher	Geographic origin	Accession number in GenBank
1	*Aristotelia fruticosa* Hook.f.	K	781	The Royal Botanic Gardens	MT982368
2	*Crinodendron patagua* Molina	K	652	The Royal Botanic Gardens	MT982369
3	*Vallea stipularis* L.f.	K	654	The Royal Botanic Gardens	MT982370
4	*Elaeocarpus angustifolius* Blume	BJFC	140942	Guangxi Academy of Forestry	MW242787
5	*Elaeocarpus hainanensis* Oliver	GNNU	PVHJX014291	Diaoluo Mountain, Hainan	MW602804
6	*Elaeocarpus japonicus* Sieb. et Zucc.	BJFC	160730004	Wugong Mountain, Jiangxi	MT985378
7	*Elaeocarpus japonicus* var. *yunnanensis* C. Chen & Y. Tang	BJFC	XW1746	Wenshan, Yunnan	MW242788
8	*Sloanea sinensis* (Hance) Hemsl.	BJFC	XW1956	Wenshan, Yunnan	MW004670
9	*Sloanea cordifolia* K. M. Feng ex H. T. Chang	BJFC	XW1958	Wenshan, Yunnan	MW242789
10	*Sloanea dasycarpa* (Benth.) Hemsl.	BJFC	XZ581	Wenshan, Yunnan	MW242790
11	*Sloanea longiaculeatae* Y. F. Xie & Z. X. Zhang	BJFC	XW1986	Wenshan, Yunnan	MW242791

**Table 2:** Species sequence numbers from the NCBI database.

Species	Accession number in GenBank	Accession number in SRA
* Aristotelia fruticosa	MT982368	SRR12599405
* Crinodendron patagua	MT982369	SRR12599428
* Elaeocarpus angustifolius	MW242787	SRR12998754
* Elaeocarpus hainanensis	MW602804	SRR13423273
* Elaeocarpus japonicus	MT683335	SRR12574443
* Elaeocarpus japonicus var. *yunnanensis*	MW242788	SRR13003726
* Sloanea cordifolia	MW242790	SRR13003865
* Sloanea longiaculeatae	MW242791	SRR13004978
* Sloanea dasycarpa	MW242790	SRR13002231
* Sloanea sinensis	MW004670	SRR12599358
* Vallea stipularis	MT982370	SRR12599429
*Averrhoa carambola*	KX364202	
*Brunellia antioquensis*	MN615725	
*Brunellia trianae*	MN585217	
*Cephalotus follicularis*	NC042597	
*Euonymus maackii*	MW771518	
*Euonymus schensianus*	NC036019	
*Oxalis corniculata*	NC051971	
*Oxalis drummondii*	NC043802	
*Rourea microphylla*	MT537171	

**Note:**

* Newly published species sequences.

### Genome annotation and comparison

The clean reads were used to assemble the chloroplast genome sequence by GetOrganelle version 1.7.7.0 with the parameters in S1 (Bankevich et al., 2012; Jin et al., 2020). A circular chloroplast genome was generated after filtered De Bruijn graphs were viewed and edited using Bandage (Wick et al., 2015; Song et al., 2017). We used Plastid Genome Annotator to annotate the assembled chloroplast genomes using as reference *E. japonicus* (MT985378; Qu et al., 2019). The annotated chloroplast genomes have been submitted to GenBank (Table 2). Schematic diagrams of all nine chloroplast genomes were drawn by the Organellar Genome DRAW tool (Lohse et al., 2013), and a map of shared protein-coding genes was drawn by a Venn diagram viewer (http://jvenn.toulouse.inra.fr/app/example.html; Philippe et al., 2014). mVISTA online tools (https://genome.lbl.gov/vista/mvista/about.shtml) were used to determine chloroplast genome similarity among Elaeocarpaceae (Frazer et al., 2004). The similarity, rearrangement and inversion of gene blocks were analyzed by Circoletto (http://tools.bat.infspire.org/circoletto/; Darzentas, 2010). IRscope (https://irscope.shinyapps.io/irapp/) was used to assess IR expansion and contraction in the evolution of chloroplast genomes (Ali, Jaakko & Peter, 2018).

### Phylogenomics and molecular clock dating analysis

To infer phylogenetic relationships within the Elaeocarpaceae and other related families, 20 species of six families including Elaeocarpaceae, Cephalotaceae, Brunelliaceae, Oxalidaceae and Connaraceae were compared. The genomes from the six families included 11 new chloroplast genomes and nine published complete chloroplast genomes (Table 2), as that of *Euonymus schensianus* (NC036019) and *Eu. maackii* (MW771518), which was obtained from the NCBI database and treated as the outgroup (Baker et al., 2021; Li et al., 2021). For the species tree, Bayesian inference (BI) analyses were performed on data sets of 20 chloroplast genome sequences. Using MAFFT version 7.490 to compare the whole genome matrix by ‘–auto’ strategy and normal alignment mode (Katoh & Standley, 2013) and then ambiguously aligned fragments were removed using Gblocks version 0.91b with the following parameter settings: minimum number of sequences for a conserved/flank position (11/11), maximum number of contiguous non-conserved positions (eight), minimum length of a block (10), allowed half of gap positions (Talavera & Castresana, 2007). Bayesian inference (BI) was performed using MrBayes version 3.2.6 (Ronquist & Huelsenbeck, 2003). The best-fitting DNA substitution model according to the Bayesian information criterion (BIC), GTR (General Time Reversible) + F (Felsenstein) + I (proportion of Invariable sites), was identified by using jModelTest version 2.1.10 (Darriba et al., 2012; Guindon, Gascuel & Rannala, 2003). Markov chain Monte Carlo simulations (MCMC) were run for 10,000,000 generations. The BI analysis started with a random tree and sampled trees every 1,000 generations. The first 25% of the trees were discarded as burn-in, and the remaining trees were used to generate a majority-rule consensus tree. Besides, we also estimated a maximum likelihood (ML) phylogeny for the genera in RAxML v8.0.0 (Stamatakis, Hoover & Rougemont, 2008), on the CIPRES web server (www.phylo.org). We used the default settings, including a TVM (Transversion model) + R3 (Free Rate three) + F (Felsenstein) model of sequence evolution. Ultrafast bootstrap with 1,000 replicates under iteration of 500 and correlation coefficient of 0.9 are used to infer the ML tree.

Then, based on BEAST 1.10.4, a lognormal distribution with an uncorrelated relaxed clock model was run by using the GTR + F + I site model with four gamma categories, with a random starting tree and a Yule speciation process tree prior (Suchard et al., 2018). MCMC was performed with 500 million generations and sampling every 50,000 generations and the effective sample size (ESS) values were confirmed exceeded 200 for all parameters. Then we used the phyutility software to generate an all-compatible consensus tree with the first 25% of the trees as burn-in (Smith & Dunn, 2008). Node ages of the consensus phylogeny were estimated using the TreeAnnotator software (Drummond & Rambaut, 2007; Dexter & Chave, 2016). A total of 95% highest posterior density intervals (HPD) for each node are shown on the tree. Additionally, the phylogeny was calibrated using four fossils, one fossil from a related clade and by setting the split between *Sloanea* and *Vallea* to 55 ± 2 Mya (Mayr, 2000). We used the 40 ± 10 Mya split between *Vallea* and *Aristotelia* as the calibration point (Heibl & Renner, 2012). *Elaeocarpus* from the Tasmania in Australia that is about 55 ± 2 Mya old (Hill, 1984). The other fossils are leaves of *Rourea* (Connaraceae) from Panama, dated to 59 Mya (Graham, 1988). The fossils are used as the ages of the nodes of the tree and applied as calibration points with the normal prior distribution. The tree was viewed and edited by FigTree version 1.4.0 software (http://tree.bio.ed.ac.uk/software/figtree/).

## Results

### Overall structure

The 11 sequenced Elaeocarpaceae chloroplast genomes showed a quadripartite structure, an LSC region, an SSC region, and a pair of IR regions, with lengths ranging from 157,546 bp (*S. sinensis*) to 159,400 bp (*C. patagua*). The length of the LSC region ranged from 85,874 bp (*E. japonicus*) to 88,413 bp (*S. sinensis*), that of the IR regions ranged from 25,984 bp (*S. sinensis*) to 27,437 bp (*E. japonicus* and *E. japonicus* var. *yunnanensis*), and that of the SSC region ranged from 16,981 bp (*E. japonicus*) to 17,958 bp (*C. Patagua*). The total GC content of the 11 chloroplast genomes from five representative genera was approximately 37%, while the GC contents of the IR, LSC and SSC regions were approximately 43%, 35% and 31%, respectively. In contrast to the chloroplast genome of *A. fruticosa*, which had 133 genes, including eight rRNA genes, 37 tRNA genes, and 88 protein-coding genes, the chloroplast genomes of the other four genera had 132 genes, including eight rRNA genes, 37 tRNA genes, and 87 protein-coding genes. A total of 114 unique genes were detected in the chloroplast genome of *A. fruticosa*, while *C. patagua*, *V. stipularis* and the genus *Sloanea* had 113 unique genes, and genus *Elaeocarpus* had 111 unique genes (Table 3).

**Table 3:** Summary of 11 complete chloroplast genomes of Elaeocarpaceae.

	*Aristotelia fruticosa*	*Crinodendron patagua*	*Vallea stipularis*	*Elaeocarpus angustifolius*	*Elaeocarpus hainanensis*	*Elaeocarpus japonicus*	*Elaeocarpus japonicus* var. *yunnanensis*	*Sloanea sinensis*	*Sloanea cordifolia*	*Sloanea dasycarpa*	*Sloanea longiaculeatae*
Total cpDNA size (bp)	158,085	159,400	158,456	158,315	157,562	157,639	158,124	157,546	158,059	157,966	157,918
Length of the LSC region (bp)	87,427	88,036	87,495	86,465	85,967	85,784	85,928	87,903	88,413	88,297	88,284
Length of the IR regions (bp)	26,477	26,703	26,615	27,038	27,135	27,437	27,437	25,984	25,985	26,011	25,985
Length of the SSC region (bp)	17,704	17,958	17,731	17,774	17,325	16,981	17,322	17,675	17,676	17,647	17,664
Total GC content	37.0%	37.0%	37.0%	36.9%	37.1%	37.1%	37.1%	37.3%	37.2%	37.2%	37.2%
GC content of the IR regions/%	42.5%	42.7%	42.4%	42.3%	42.3%	42.2%	42.2%	42.9%	42.9%	42.9%	42.9%
GC content of the LSC region/%	34.9%	34.7%	34.9%	34.8%	34.9%	35.0%	34.9%	35.1%	35.0%	35.1%	35.0%
GC content of the SSC region/%	30.9%	30.8%	30.9%	31.0%	31.2%	31.3%	31.2%	31.4%	31.3%	31.4%	31.3%
Total number of genes (unique)	133 (114)	132 (113)	132 (113)	132 (111)	132 (111)	132 (111)	132 (111)	132 (113)	132 (113)	132 (113)	132 (113)
Protein-encoding genes	88	87	87	87	87	87	87	87	87	87	87
tRNAs	37	37	37	37	37	37	37	37	37	37	37
rRNAs	8	8	8	8	8	8	8	8	8	8	8

### Chloroplast genome comparisons

**Figure 1:**
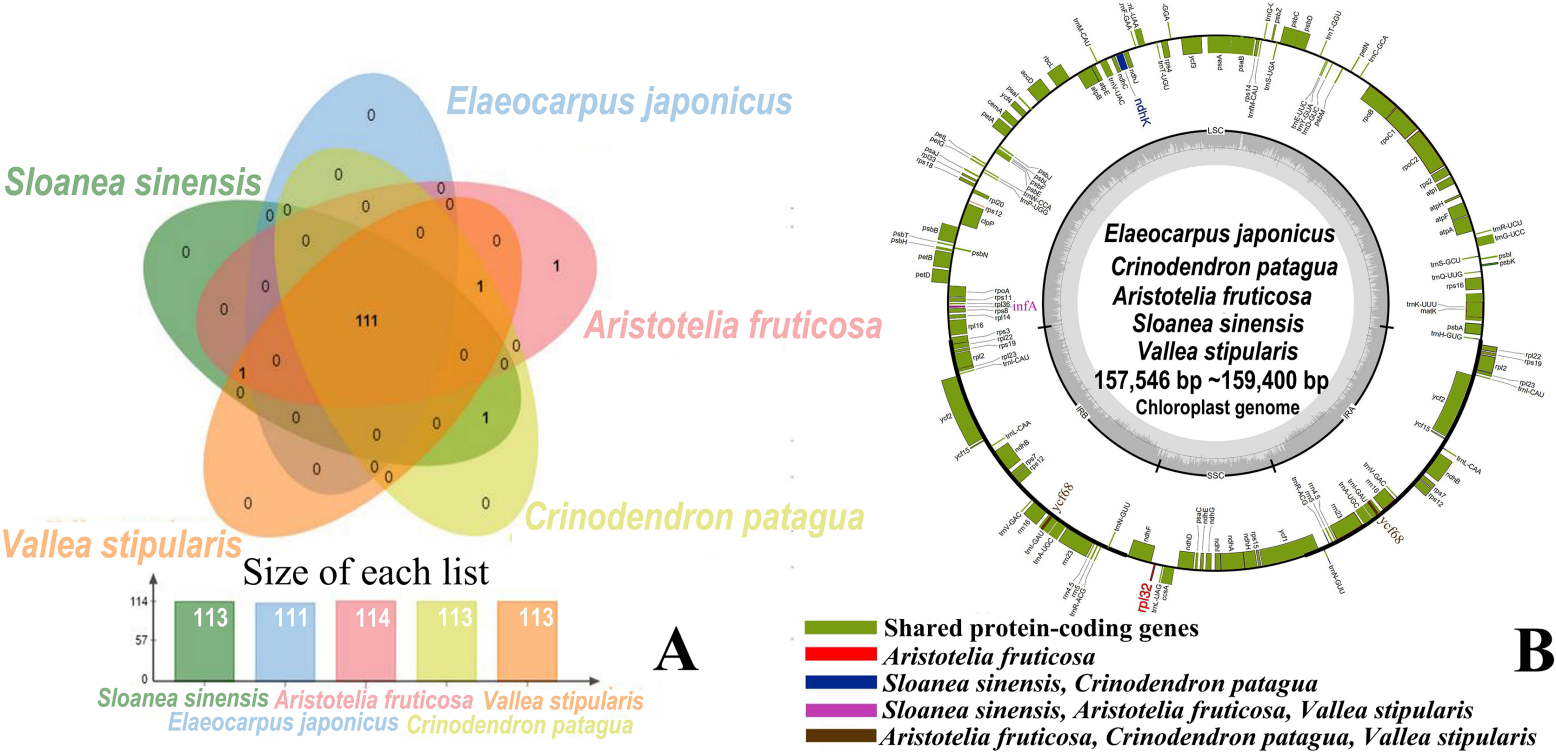
Shared protein-coding genes in Elaeocarpaceae chloroplast genomes. (A) Shared protein-coding genes in Elaeocarpaceae chloroplast genomes. The Venn diagram illustrates the number of genes shared between the chloroplast genomes of *Aristotelia fruticosa*, *Crinodendron patagua*, *Vallea stipularis*, *Elaeocarpus japonicus* and *Sloanea sinensis*. (B) Chloroplast genome map of *Aristotelia fruticosa*, *Crinodendron patagua*, *Vallea stipularis*, *Elaeocarpus japonicus* and *Sloanea sinensis*. The green block represents shared protein-coding genes. The red block represents the genes unique to *Aristotelia fruticosa*. The blue block represents the genes unique to *Sloanea sinensis* and *Crinodendron patagua*. The pink block represents the genes unique to *Sloanea sinensis*, *Aristotelia fruticosa* and *Vallea stipularis*. The brown block represents the genes unique to *Aristotelia fruticosa*, *Crinodendron patagua* and *Vallea stipularis*.

**Figure 2:**
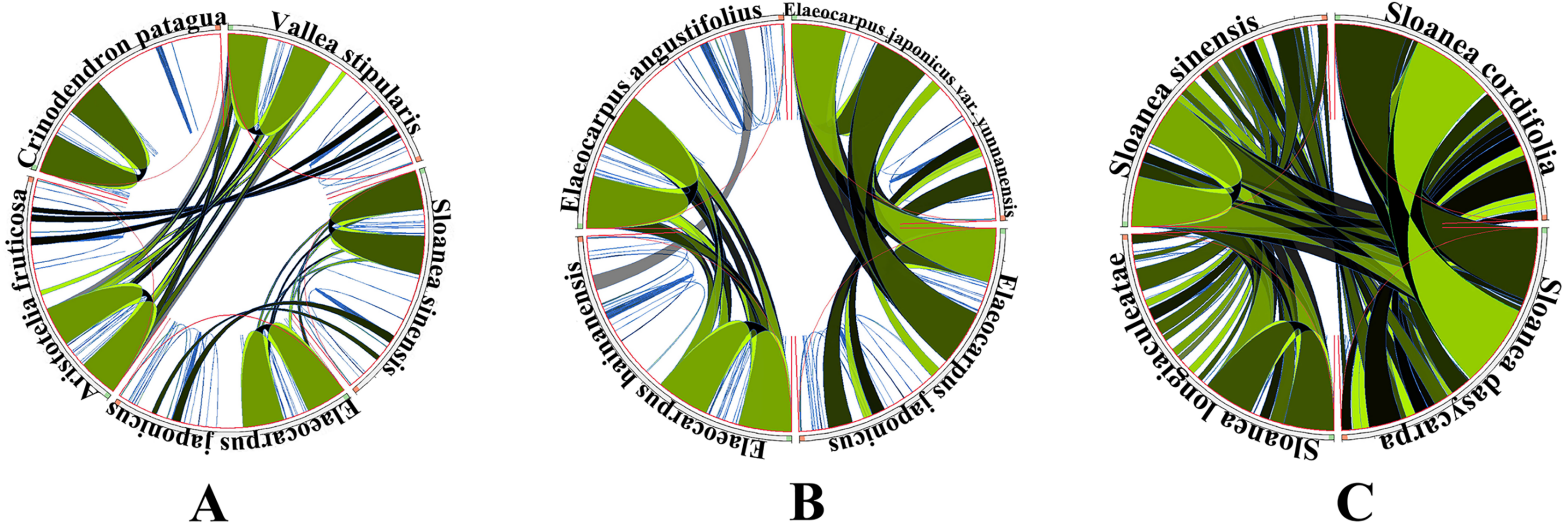
Synteny detected in Elaeocarpaceae using Circoletto. (A) Synteny detected between the chloroplast genomes of the Elaeocarpaceae species *Aristotelia fruticosa*, *Crinodendron patagua*, *Vallea stipularis*, *Elaeocarpus japonicus* and *Sloanea sinensis* using Circoletto. (B) Synteny detected between the chloroplast genomes of the Elaeocarpaceae species *Elaeocarpus angustifolius, Elaeocarpus japonicus, Elaeocarpus japonicus* var. *yunnanensis* and *Elaeocarpus hainanensis* using Circoletto. (C) Synteny detected between the chloroplast genomes of the Elaeocarpaceae species *Sloanea cordifolia, Sloanea dasycarpa, Sloanea longiaculeatae* and *Sloanea sinensis* using Circoletto.

The five genera shared 111 protein-coding genes, but *rpl*32 was only detected in the SSC region of the chloroplast genome of *A. fruticosa* (Fig. 1). The LSC region of the chloroplast genomes lacked *ndh*K in the genus *Elaeocarpus*, *V. stipularis*, and *A. fruticosa* and lacked *inf*A in the genus *Elaeocarpus* and *C. patagua*. *ycf*68 was found in *V. stipularis*, *A. fruticosa* and *C. patagua*. In addition, synteny was detected in the five genera of Elaeocarpaceae (Fig. 2). A significant degree of synteny was found between *V. stipularis* and *A. fruticosa*, *Elaeocarpus* and *Sloanea*. However, the synteny between *C. patagua* and the other four genera was low. Five genera of Elaeocarpaceae were compared, in addition to the species in *Elaeocarpus* and *Sloanea*. Two groups, *E. angustifolius* and *E. hainanensis* as well as *E. japonicus* and *E. japonicus* var. *yunnanensis*, had more blocks of synteny in the genus *Elaeocarpus*. Several blocks of synteny were detected in the four chloroplast genomes of the genus *Sloanea*, suggesting that the four species are similar to each other.

### IR expansion and contraction

**Figure 3:**
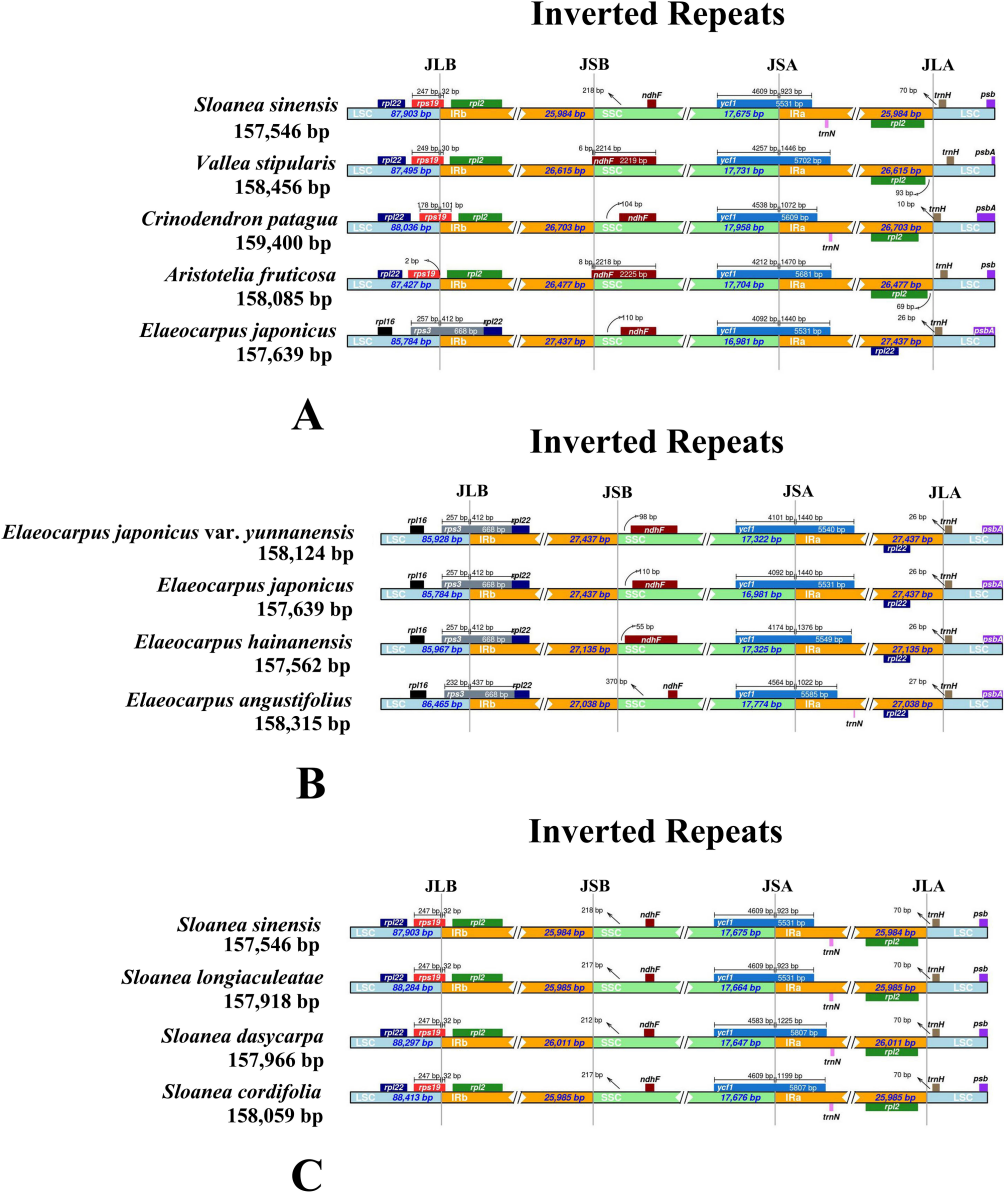
Comparisons of IR expansion and contraction in Elaeocarpaceae. (A) The chloroplast genome boundaries of *Aristotelia fruticosa, Crinodendron patagua, Vallea stipularis, Elaeocarpus japonicus* and *Sloanea sinensis* of Elaeocarpaceae. (B) The chloroplast genome boundaries of *Elaeocarpus angustifolius*, *Elaeocarpus japonicus, Elaeocarpus japonicus* var. *yunnanensis* and *Elaeocarpus hainanensis*. (C) The chloroplast genome boundaries of *Sloanea cordifolia, Sloanea dasycarpa, Sloanea longiaculeatae* and *Sloanea sinensis*.

In the sequenced chloroplast genomes of Elaeocarpaceae, two complete or fragmented copies of *rps*19 and *rpl*2 were located at the boundaries between the LSC region and IRa or IRb region in *V. stipularis*, *A. fruticosa*, *C. patagua* and the genus *Sloanea* (Fig. 3). In contrast, *rps*3, *rpl*22 and *rpl*16 were detected in the neighboring regions of the LSC or IRa or IRb region in the genus *Elaeocarpus*. The distance between the fragment of *ndh*F and the boundary of the SSC and IRb regions in *E. angustifolius* was 370 bp, much greater than that in the chloroplast genomes of other species in the genus *Elaeocarpus*: *E. japonicus* var. *yunnanensis*, *E. japonicus* and *E. angustifolius*. Moreover, the lengths of *ndh*F and *ycf*1 in *E. angustifolius* were shorter than those in the other three species. For the genus *Sloanea*, the chloroplast genomes of four species, *S. sinensis*, *S. cordifolia*, *S. dasycarpa* and *S. longiaculeatae*, were generally the same in terms of IR expansion and contraction, with the exception that the length of *ycf*1 in *S. dasycarpa* and *S. longiaculeatae* was greater than that in *S. sinensis* and *S. cordifolia*.

### Phylogenomics and molecular clock dating analysis

**Figure 4:**
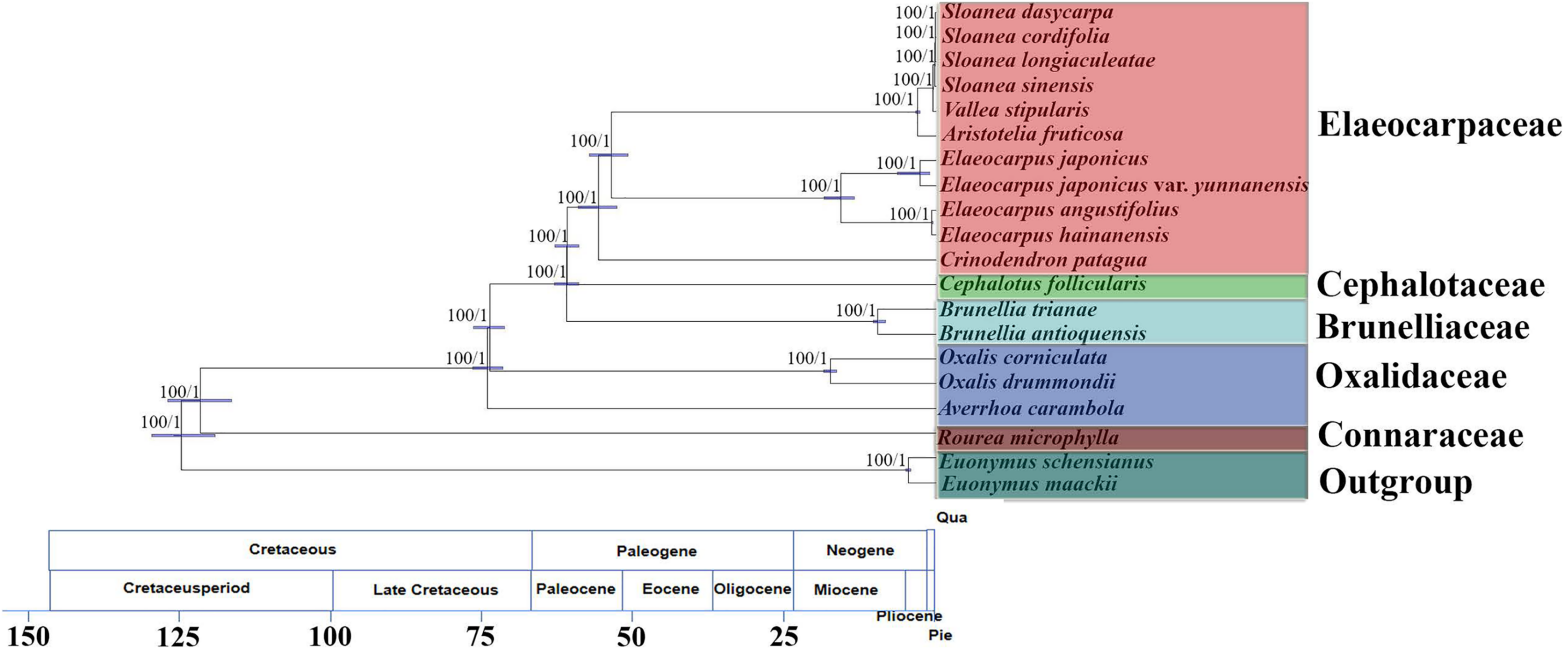
Molecular phylogenomic tree of 20 species of Oxalidales. Molecular phylogenomic tree of 20 species of Oxalidales based on complete chloroplast genome sequences constructed using Bayesian Inference (BI) and Maximum Likelihood (ML). Numbers at each node are bootstrap support values and posterior probability. The blue bars indicate 95% highest posterior density intervals (HPD) of the age estimate. Divergence time of clades and subclades are displayed on the bottom of picture.

The matrix of complete chloroplast genomes was used to reconstruct a phylogenomic tree of Oxalidales (Fig. 4). The phylogenetic tree constructed by the ML analysis is consistent with the phylogenetic tree based on BI method. The phylogenies show high robustness with the highest posterior probability and bootstrap value for all clades. The molecular tree showed that *R. microphylla* representing Connaraceae started to diversify at 119 Mya (95% highest posterior probability density intervals (HPD):114–125 Mya). *Averrhoa carambola*, *Oxalis drummondii* and *Ox. corniculata* representing Oxalidaceae diverged from basal Oxalidales (ca. 122 Mya) at 73 Mya (HPD:70–75 Mya). Cephalotaceae (ca. 60 Mya; HPD:58–62 Mya) has the closest genetic relationship with Elaeocarpaceae (ca. 60 Mya; HPD:58–62 Mya) with that of Oxalidaceae (ca. 119 Mya; HPD:114–125 Mya). Brunelliaceae has a similar differentiation time about 60 Mya (HPD:58–62 Mya) from Oxalidaceae.

The molecular tree also showed the sister relationships of 11 chloroplast genomes from five representative genera of Elaeocarpaceae were highly supported. Clade I, containing the genus *Elaeocarpus*, was 100% supported and was dated to ca. 53 Mya (HPD:50–56 Mya), and the crown node age of clade II (*C. patagua*) was dated to ca. 55 Mya (HPD:52–58 Mya). Diversification of clade III, containing the *Sloanea* alliance (*V. stipularis*, *A. fruticosa* and the genus *Sloanea*), was dated to 53 Mya (HPD:50–56 Mya). Further differentiation of *V. stipularis* and *A. fruticosa* took place within the last 3 Mya (HPD:2–3 Mya). In addition, the genus *Sloanea* started to diversify during the late Miocene (ca. 0.4 Mya; HPD:0.3–0.4 Mya).

## Discussion

### Complete chloroplast structure of Elaeocarpaceae

This study included 11 complete chloroplast genomes for Elaeocarpaceae plants. All these complete chloroplast genomes had a total GC content of 37%, consistent with the low GC content in the chloroplast genomes of other angiosperms. The higher the content of GC is, the higher the density of DNA and the more conserved the chloroplast genome is Do, Kim & Kim (2013). Therefore, variation might occur in the SSC region rather than the IR regions. Comparisons of the 11 plastomes showed the loss of *inf*A in *C. patagua* and the genus *Elaeocarpus*, and similar losses or pseudogenization was reported in the 309 complete chloroplast genomes of 24 species of angiosperms (Millen et al., 2001). *ndh* genes are frequently pseudogenized or lost in plant groups with a degree of heterotrophy due to evolutionary adaptation to excessive water in the environment, as observed in *Aneura*, *Cuscuta*, *Epifagus*, *Hydnora*, and nonphotosynthetic orchid species and some autotrophic gymnosperms and ferns (DePamphilis & Palmer, 1990; McNeal et al., 2007; Wickett et al., 2008; Wicke et al., 2011; Kim et al., 2015; Naumann et al., 2016). The *rpl*32 gene was detected in *A. fruticosa* but not in the other four genera (*V. stipularis*, *C. patagua*, the genus *Elaeocarpus* and the genus *Sloanea*), which is similar to previously published research about the losses of two genes, *inf*A and *rpl*32, in *Thalictrum coreanum* (Park & Jansen, 2015). In summary, the five genera may have experienced different niche expansions.

Previous studies have shown that IR boundary regions with large expansions and contractions may be related to double-strand breakage and repair, while small expansions and contractions may be related to gene conversions, which is a common phenomenon in the evolution of the chloroplast genomes (Kim & Lee, 2004; Khakhlova & Bock, 2006; Hansen et al., 2007; Wang et al., 2008; Ma et al., 2013; Liang, Wen & Gao, 2018). We found large IR expansions in the five genera. The genus *Elaeocarpus* is different from the other four genera at the IR/SC boundary, which may reflect that the genus *Sloanea* has an older origin and experienced a different evolution event. In addition, *rps*19 was located across the LSC/IRB regions in four genera, while the boundary of the LSC and IRb regions in the genus *Elaeocarpus* included *rps*3. Research shows that the locations of *rps*19 and *rps*3 differ between the chloroplasts of monocotyledons and dicotyledons. In some dicotyledons, *rps*19 only partially exists in the IR region, while the *rps*3 gene is only found in *Paris* and Melanthiaceae (Lin et al., 2012; Sarah, Kim & Kim, 2013). Compared with the other four genera, the genus *Sloanea* experienced different complex evolutionary events.

Homologous fragments have been found *via* collinearity analysis in various plants, including Capparaceae (Alzahrani et al., 2021), Ranunculaceae (Park & Park, 2021), and *Passiflora* (Cauz-Santos et al., 2020). The length of homologous fragments is related to the time of divergence between species. The shorter the time of species differentiation is, the more homologous fragments there are (Cheng et al., 2013). According to the similarity of the 11 chloroplast genomes of Elaeocarpaceae, we detected several blocks of synteny between *V. stipularis* and *A. fruticosa*, the genus *Elaeocarpus* and the genus *Sloanea*, meaning that the times of divergence between the genus *Sloanea* and the genus *Elaeocarpus*, *V. stipularis* and *A. fruticosa* was similar. Interestingly, there were no blocks of synteny in *C. patagua* with the other four genera, meaning that the evolution of *C. patagua* was different from the rest of the genera. In the genus *Elaeocarpus* and genus *Sloanea*, it is worth noting that the divergence time of *E. japonicus* was similar to that of *E. japonicus* var. *yunnanensis* and that of *E. angustifolius* was similar to that of *E. hainanensis*. In addition, the times of divergence among species in the genus *Sloanea* were similar.

### Phylogenomic relationships and historical biogeography in Oxalidales

Based on the 20 species of six families with available complete chloroplast genomes, a phylogenomic tree of Oxalidales was reconstructed, consistent with the recent phylogeny (Byng et al., 2016; Baker et al., 2021; Li et al., 2021). Elaeocarpacea was clarified as sister to Cephalotaceae and Brunelliaceae and Connaraceae. In addition, Oxalidaceae is far from Elaeocarpaceae as previously recognized, which was recognized by Heibl & Renner (2012).

Pillon et al. (2021) phylogeny of Oxalidales based on plastid genomics has been used as data for event-based biogeographic analysis of the world. In that study the likely ancestral area for the Oxalidales is Australia/New Guinea + New Caledonia in Cretaceous, which was consistent with our results. Indeed, the greatest number of extant species and genera are found in Oceania, and particularly in eastern Australia, New Guinea, and New Caledonia (Kershaw, 1976; Kershaw, Bretherton & van der Kaars, 2007; Sniderman, 2011).

The age of the Connaraceae clade with *R. microphylla* was much older than previously estimated (74 Mya; Heibl & Renner, 2012). The recent discovery of *Connarus*-like wood from the Paleocene of India, outside the modern range of the family, suggests a possible origin in India during the Cretaceous, when India was an island continent, and subsequently spread throughout the Old World tropics as India docked with Asia (Baas et al., 2017).

The differentiation time of Oxalidaceae is consistent with that of Heibl and Renner, which is about 68 Mya. Geographical distribution patterns suggest the origin of the family in the southern hemisphere, prior to the separation of South America and Africa (Raven & Axelrod, 1974).

The split from Cephalotaceae and Brunelliaceae was estimated at 60 Mya, more recent than Heibl and Renner’s research (78 Mya). According to recent research, *Brunellia* is exclusively American, with only six of the 61 known species occurring north of Panama. Cephalotaceae grows only in the extreme SouthWest of Australia (Matthews & Endress, 2002). The presence of Brunelliaceae and Cephalotaceae may indicate that the genera may have been represented north of Panama before the closing of the central American land bridge (Coode, 2004).

As for the differentiation time of Elaeocarpaceae, it has long been postulated that the family Elaeocarpaceae originated in the southern hemisphere, of which only *Elaeocarpus* and *Sloanea* reach the northern hemisphere (Raven & Axelrod, 1974). The crown age of Elaeocarpaceae estimated in this study based on molecular data was younger than the age previously estimated at 79.62–85.2 Mya (Magallon et al., 2015; Phoon, 2015), 64–66 Mya (Wikström, Savolainen & Chase, 2001), 67 Mya (Heibl & Renner, 2012) and 100 Mya (Crayn, Rossetto & Maynard, 2006), older than 38 Mya (Harris & Davies, 2016). These differences may be due to the choice of DNA markers and the accuracy of the fossil calibrations of molecular evolutionary rates. The earliest divergence within the Elaeocarpaceae appears to have occurred in the late Cretaceous based on our data, which is broadly coincident with the time when the western (Africa and South America) and eastern (Australia, Antarctica, Madagascar and India) parts of Gondwana were separating (Ali & Aitchison, 2008).

### Phylogenomic relationships and historical biogeography in Elaeocarpaceae

Within Elaeocarpaceae, the 11 taxa were separated into the following groups in our study: the *Sloanea* alliance (*V. stipularis*, *A. fruticosa* and *Sloanea*), *Elaeocarpus* alliance and *C. patagua* alliance. The phylogenomic placements are consistent with those in Phoon’s research (Phoon, 2015). One major challenge in previous studies of the phylogenetic relationships between and within Elaeocarpaceae was the focus on DNA markers (*trn*L-*trn*F region and *trn*V-*ndh*C region) rather than complete chloroplast genomes (Maynard, 2004; Baba, 2013; Phoon, 2015). Furthermore, the DNA markers exhibited low sequence variability, leading to insufficiently resolved phylogenies within *Elaeocarpus*, and there no phylogenetic tree was constructed for *Sloanea*. Our phylogenomic analysis strongly confirmed the preliminary results of previous studies but with higher robustness, and the results of the analysis improved the posterior probabilities of all clades (Maynard, 2004; Baba, 2013; Phoon, 2015).

Compared with the differentiation of *V. stipularis* and *A. fruticosa* in Phoon’s study, the age of the split between *V. stipularis* and *A. fruticosa* was much younger than previously estimated at 37 Mya (Phoon, 2015). The results of the present study agreed with Coode’s phylogenetic reconstruction in which *Vallea* and *A*. were sisters and Phoon’s finding that the ancestors may have dispersed between western and eastern Gondwana. The minimum estimates of divergence times between *V. stipularis* and *A. fruticosa* because the divergence of the South American and New Zealand lineages at 24–27 and 3 Mya respectively, postdates the isolation of their respective landmasses (McLoughlin, 2001).

*Crinodendron* was resolved in this study as an independent branch. The split from Elaeocarpaceae was estimated at 55 Mya, more recent than Phoon’s estimate (59 Mya). The divergence of *Crinodendron* is estimated to have occurred during the Paleo-Eocene, but the origin of the genus is almost certainly older given the position of *Dubouzetia brasiliense* (from a dwarf cloud forest near the Atlantic coast of Brazil) as sister to the rest of the genus, based on morphological data (Coode, 2004).

*Elaeocarpus* represents a widespread lineage in Elaeocarpaceae that diverged 53 Mya, which was older than Phoon’s estimate (40 Mya). Divergence time analysis suggests that *Elaeocarpus* split in the Eocene and migrated out of Australia to the surrounding regions mostly in the Oligocene and the Miocene, although the taxon sampling without species of Southeast Asian in this clade has led to these dates being doubted (Crayn, Rossetto & Maynard, 2006; Heibl & Renner, 2012; Phoon, 2015).

Divergence time analysis suggests that *Sloanea* diverged from its sister species *V. stipularis* and *A. fruticosa* at 0.4 Mya, more recent than Phoon’s estimate (29 Mya). The reason may be that *Sloanea* in China belongs to a branch of the *Slonaea* genus, and the differentiation time is late.

Overall, the divergence times of all genera in Elaeocarpaceae inferred using the complete chloroplast genomes were more accurate than those inferred using DNA markers (*trn*L-*trn*F region and *trn*V-*ndh*C region).

## Conclusions

In the present research, the chloroplast genomes of nine species were assembled using Illumina high-throughput sequencing data. The genomic structures of the 11 samples were compared and analyzed. On this basis, we concluded that the chloroplast genome structure and gene size in Elaeocarpaceae showed some difference. In addition, we determined the relationships of Elaeocarpaceae within Oxalidales and inferred divergence dates and the biogeographic history of the major clades within Oxalidales and Elaeocarpaceae. These results are consistent with previous studies reporting relationship in Elaeocarpaceae and provide new insight into the evolution of the Elaeocarpaceae.

## Supplemental Information

10.7717/peerj.15322/supp-1 Supplemental Information 1 Parameters in Cutadapt, Trimmomatic and Getorganelle.

## Additional Information and Declarations

### Data Availability

The following information was supplied regarding data availability: The data are available at GenBank: MT683335, MW004670, MT982369, MW602804, MW242791, MW242788, MW242787, MW242790, MT982370, MW242790, MT982368.
