# Supplementary material for: Comparison of the chloroplast genomes and phylogenomic analysis of Elaeocarpaceae
Source: PeerJ. 2023 May 9;11:e15322. doi: 10.7717/peerj.15322 (PMC10178313; doi:10.7717/peerj.15322)
Supplement: Supplemental Information 1 [file peerj-11-15322-s001.docx]

**Supplementary 1**

1. Cutadapt parameters: --times 1 -e (maximum allowed error rate) 0.1 -O (overlap) 3 --quality-cutoff 25 -m (minimum length) 45

Cutadapt key code: cutadapt -a XXXXXXXXX -A XXXXXXXX --times 1 -e 0.1 -O 3 -m 45 --quality-cutoff 25 --pair-filter=both -o E1_1_trimmed.fastq.gz -p E2_2_trimmed.fastq.gz E1_1.fastq.gz E2_2.fastq.gz

1. Trimmomatic parameters: LEADING (cut bases off the start of a read, if below a threshold quality):28 TRAILING (cut bases off the end of a read, if below a threshold quality):28 SLIDINGWINDOW (perform a sliding window trimming, cutting once the average quality within the window falls below a threshold):5:15 MINLEN (drop the read if it is below a specified length):50

Trimmomatic key code: java -jar /home/wangyihui/miniconda3/share/trimmomatic-0.39-2/trimmomatic.jar PE -threads 2 -phred33 E1_1_trimmed.fq E2_2_trimmed.fq Elaeocarpus_1_trimmed_clean.fq Elaeocarpus_2_trimmed_clean.fq ILLUMINACLIP:/home/wangyihui/miniconda3/share/trimmomatic-0.39-2/adapters/TruSeq3-PE.fa:2:30:10 LEADING:28 TRAILING:28 SLIDINGWINDOW:5:15 MINLEN:50

1. Getorganelle parameters：-w (the value word size) 0.6 -R (maxium extension rounds) 15 -k (SPAdes kmer settings) 45,65,85,105,125 -F (target organelle genome type) embplant_pt.

Getorganelle key code: get_organelle_from_reads.py -1 Elaeocarpus_1_trimmed_clean.1.fq.gz -2 Elaeocarpus_2_trimmed_clean.2.fq.gz -w 0.6 -t 1 -o Elaeocarpus_simulated.plastome -F embplant_pt -R 15 -k 45,65,85,105,125
